# Comparison of health economics in robot-assisted partial nephrectomy and CT-guided cryoablation for the management of T1 renal cell carcinoma: an analysis of a prospective Danish cohort

**DOI:** 10.1007/s00270-025-04224-2

**Published:** 2025-10-15

**Authors:** Theresa Junker, Mie Gaedt Thorlund, Tommy Kjærgaard Nielsen, Nessn Azawi, Signe Wang Bach, Jonathan Belsey, Jens Borgbjerg, Ole Graumann

**Affiliations:** 1 Department of Radiology, Aarhus University Hospital, Aarhus, Denmark; 2 Research and Innovation Unit of Radiology - UNIFY, University of Southern Denmark, Odense, Denmark; 3 Department of Urology, Odense University Hospital, Odense, Denmark; 4 Department of Urology, Aalborg University Hospital, Aalborg, Denmark; 5 Department of Clinical Medicine, Aalborg University, Aalborg, Denmark; 6 Clinical Cancer Research Center, Aalborg University Hospital, Aalborg, Denmark; 7 Department of Urology, Zealand University Hospital, Roskilde, Denmark; 8 Department of Urology, Copenhagen University Hospital, Herlev and Gentofte Hospital, Copenhagen, Denmark; 9 JB Medical Ltd, London, UK; 10 Department of Radiology, Akershus University Hospital, Lørenskog, Norway; 11 Department of Clinical Medicine, Aarhus University, Aarhus, Denmark

**Keywords:** Renal cell carcinoma, Partial nephrectomy, Focal cryoablation, Cost-effectiveness analysis, Focal therapy, Propensity score matching, Objective world evidence

## Abstract

**Purpose:**

This study used real-world outcomes data to compare the cost-effectiveness of percutaneous cryoablation (PCA) and robot-assisted partial nephrectomy (RAPN) in patients with T1 renal cell carcinoma (RCC).

**Materials and Methods:**

Prospective data from June 2019 to February 2021 from two Danish University hospitals, following patients with RCC stage T1 treated with either PCA or RAPN, were used to provide procedural and clinical outcome parameters. A Markov model was used to estimate quality-adjusted life years (QALYs) and costs, incorporating health states for stable disease, local recurrence, metastasis, and all-cause mortality. Propensity score matching using specific covariates was carried out to ensure that the two populations evaluated were matched. Analyses were conducted comparing time to local recurrence or metastases, duration of hospital stay, and postoperative complications. Treatment-specific mortality was not included in the model due to the low number of deaths observed.

**Results:**

There were no significant differences between PCA and RAPN in terms of local recurrence (HR = 0.80; 95% CI = 0.34–1.85; *p* = 0.72), metastases (HR = 2.09; 95% CI = 0.69–6.26; *p* = 0.19), or Clavien-Dindo III + complications (5.5% vs 2.5%; *p* = 0.325). There were significant differences in the mean duration of hospital stay (1.13 days versus 1.90 days; *p* < 0.001). QALYs gained were nearly identical for each treatment; however, PCA was associated with a net monetary benefit of €9,045 at a willingness-to-pay threshold of €40,000/QALY.

**Conclusion:**

The present study suggests that PCA could equally benefit patients with RCC T1 by providing cost savings, making it a more cost-effective treatment without compromising oncological outcomes.

**Level of evidence:**

2b, Analysis based on clinically sensible costs or alternatives, including multi-way sensitivity analyses.

**Graphical Abstract:**

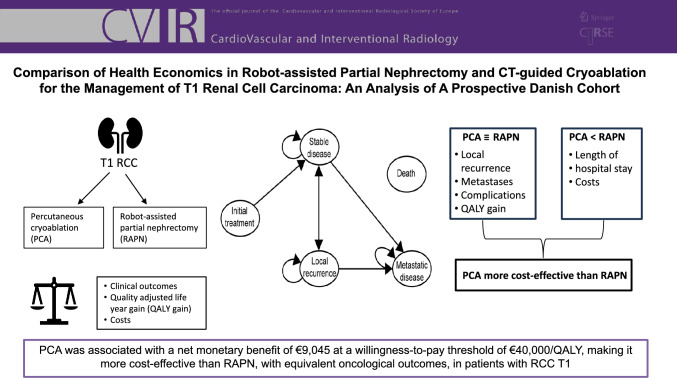

## Introduction

Renal cell carcinoma (RCC) accounts for 90–95% of all renal malignancies. In 2020, there were around 400,000 incident cases and 130,000 deaths globally [1, 2]. A 2019 publication estimated that new cases of RCC in Denmark would rise from 922 in 2020 to 1,017 by 2030 [3]. In 2022–2023, the Danish Renal Cancer database documented 1,091 new cases, suggesting that the rise in incidence has already exceeded the predicted rate for 2030 [4].

A 5-year survival following diagnosis of RCC ranges from 97% for stage *I to 27%* for stage IV [5]. Effective containment of stage *I* tumors, around two-thirds of all new cases [5], is a high therapeutic priority. The standard treatment for early-stage localized RCC is robot-assisted partial nephrectomy (RAPN) [6, 7] to maximize the preservation of renal parenchyma without sacrificing cancer control.

Percutaneous cryoablation (PCA) is an alternative method in which cryoprobes are inserted into the tumor under computed tomography (CT) guidance. A double freeze–thaw cycle destroys the tumor cells through intracellular ice formation, osmotic cell membrane rupture, and delayed local vascular injury. This approach is associated with oncological outcomes similar to RAPN’s regarding local recurrence, metastatic disease, and cancer-specific mortality, with a low complication rate [8–10].

Current guidelines from the European Association of Urology and the American Urological Association recommend PCA as an alternative to RAPN in patients with T1a tumors, particularly in those with frailty or comorbidities [6, 7]. This restricted range of indications reflects the relatively limited published evidence base for PCA.

PCA is currently used in a minority of patients with RCC; however, a recent analysis from Germany and the USA shows that its use grew threefold between 2006 and 2019 [11]. Drivers for adopting PCA were improved safety and a shorter procedure time than other focal therapies [12]. While primarily seen in older patients, 15% of PCA patients in the German analysis were under 65, indicating an overlap with the PN target group [11].

One aspect of PCA that merits consideration is the relative cost performance versus RAPN. While long-term costs are likely similar, the costs of the procedure and the postoperative care can vary considerably. However, potentially significant differences between the patient populations undergoing the two treatments could impact the analysis and bias the conclusions. Several recent studies have explored the costs of PCA versus surgical treatment of T1a RCC**,** with mixed results depending on methodology, healthcare setting, and inclusion criteria [12–15]. However, the evidence is sparse, especially prospective data regarding the cost-effectiveness of PCA versus RAPN in a publicly funded healthcare system, including both T1a and T1b tumors with long-term follow-up. Therefore, this study used real-world prospective clinical data from two centers in Denmark to assess the cost-effectiveness of PCA compared to RAPN in patients with stage T1 RCC, using propensity score matching to allow assessment of the impact of the treatment modality independently of patient characteristics.

## Methods

The objective was to compare patients with cT1N0M0 RCC undergoing primary treatment, using either RAPN or PCA, as part of a prospective cohort study in two Danish university hospitals. A Markov model was constructed in TreeAge Pro (TreeAge Software, 2024) to estimate medium-term quality-adjusted life years (QALYs) and direct medical costs from the perspective of the Danish healthcare system over a 5-year time horizon. QALYs and costs were discounted at 3% per year. The Danish Data Protection Agency approved the project and was deemed exempt from ethics notification obligations. The study was reported following the CHEERS guidelines [16]

### Model Structure

**Fig. 1:**
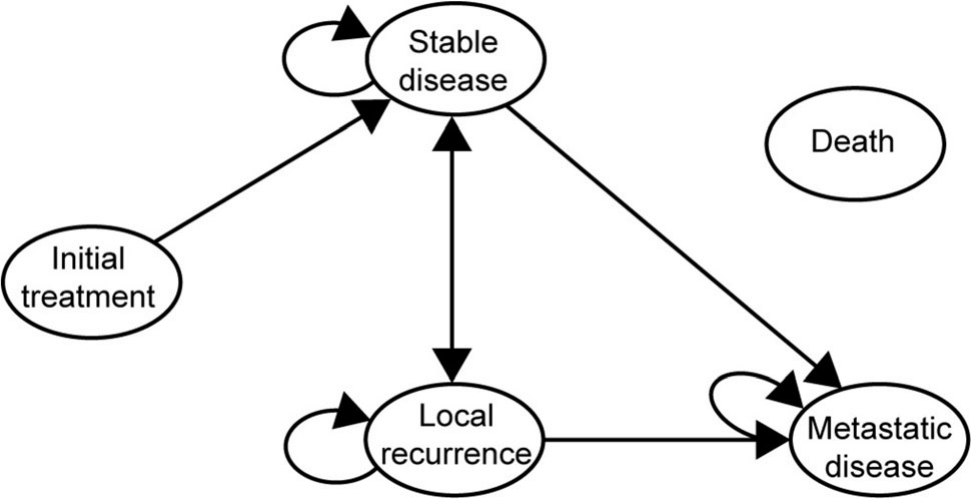
Health state transition diagram

**Fig. 2:**
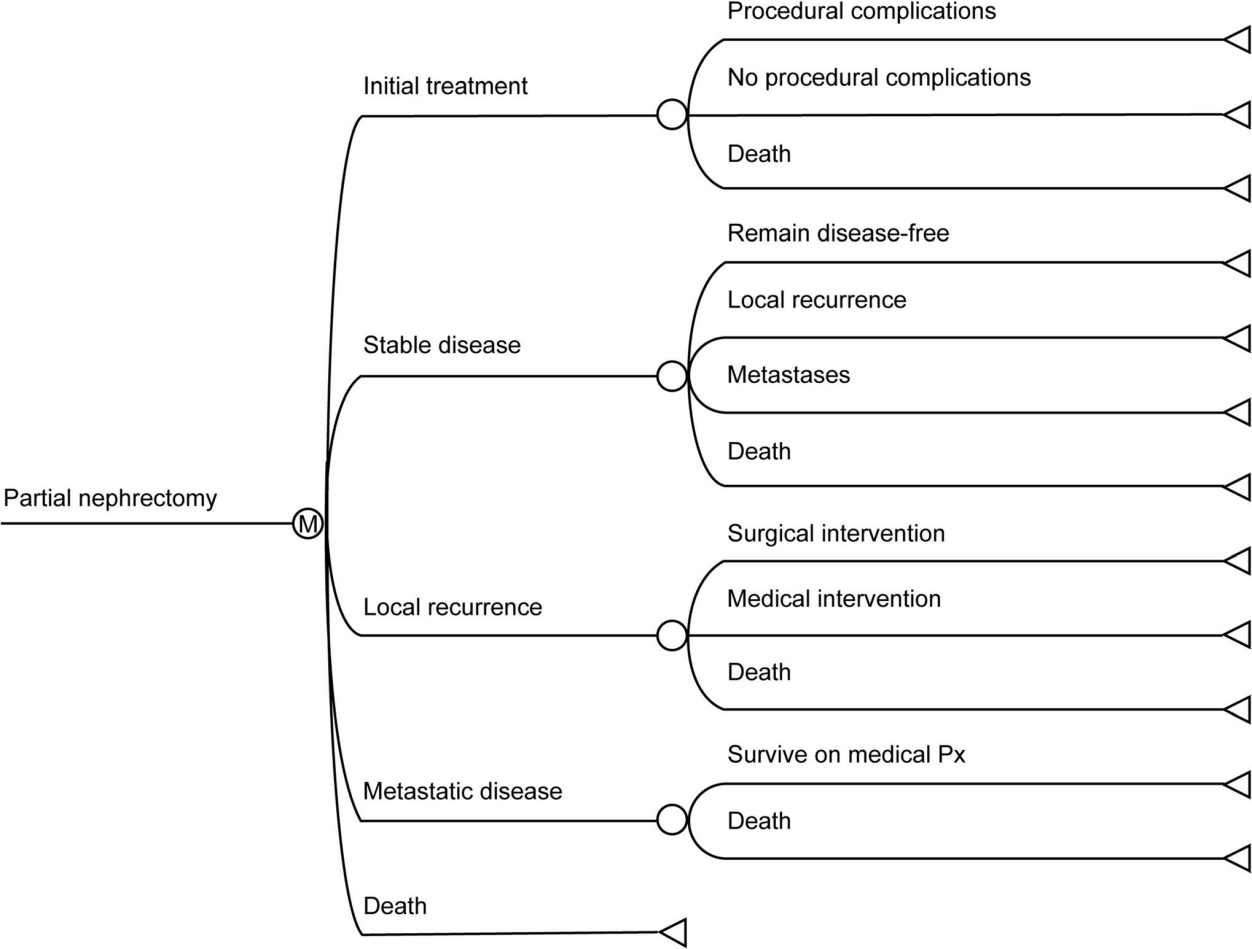
Cost-utility model structure (partial nephrectomy shown)

The Markov model consisted of three post-treatment health states (stable disease, local recurrence, and metastatic disease) and a further state capturing death (Figs. 1 and 2).

### Input Parameters

Table 1 shows the input parameters and sources used. All variables were included in a deterministic sensitivity analysis, with testing being carried out across a range of ± 20% from the central estimate.

**Table 1:** Input parameters used in the model

Parameter	Value	Source
*Transition probabilities*		
Postoperative complications: RAPN (Clavien grade III +)	2.5%	Analysis of NEPHSPARE data^*^
Postoperative complications: PCA (Clavien grade III +)	5.5%	Analysis of NEPHSPARE data^*^
Local recurrence	0.18% per month	Weighted analysis of NEPHSPARE data^*^ Same rate applied in both groups
Metastatic recurrence	0.35% per month	Weighted analysis of NEPHSPARE data^*^ Same rate applied in both groups
Overall mortality	0.12% per month	Weighted analysis of NEPHSPARE data^*^ Same rate applied in both groups
Mortality in metastatic state	Parameterised survival curve	Hawkins et al. [19] – survival following targeted systemic therapy in metastatic RCC
*Utilities*		
Intervention	0.058 in month of procedure	Wu et al. [13] (assumes local recurrence state for month of treatment)
Postoperative complications	0.05 in month of procedure	Wu et al. 2023 [13]
Stable disease	0.76	Wu et al. 2023 [13]
Local recurrence	0.70	Wu et al. 2023 [13]
Metastatic recurrence	0.66	Wu et al. 2023 [13]
*Health care resource use & costs*		
Cost of PCA procedure	€7,427	Bottom-up costing based on clinician input
Cost of RAPN procedure	€15,293	Health Data Agency DRG Rates 2024: DRG 11MP07
Cost of post-op complications	€4,332	Health Data Agency DRG Rates 2024: DRG 21MA03
Cost/month of advanced disease state	€2,500	Estimated based on published literature Buja et al.; Ronconi et al.; Cholley et al.; Redig et al. [30–33]

^*^Data from the NEPHSPARE cohort have previously been published regarding adverse events and health-related quality of life [17, 18]

### Clinical Inputs

Procedural and clinical outcomes were derived from the results based on the NEPHSPARE cohort [17, 18]. This prospective cohort of 187 patients with cT1N0M0 RCC underwent primary treatment between June 2019 and February 2021 at two university hospitals in Denmark. All PCA procedures were conducted at one hospital specializing in the technique. Both centers performed RAPN. The present analysis is on a subset of 171 patients from the NEPHSPARE cohort, excluding open partial nephrectomy (PN). The median duration of follow-up was 30 months. Patients were treated with either RAPN (*n* = 80) or PCA (*n* = 91), as decided by a multidisciplinary team. PCA patients tended to be older and have smaller tumors, more comorbidities, poorer renal function, and a lower performance status than those treated with RAPN (Table 2).

**Table 2:** Covariate distributions in crude population and propensity-adjusted pseudopopulation

Covariate	Crude population			IPTW-adjusted population			
	PCA	RAPN	*p*	PCA	RAPN	*p*	
Number of patients	91	80		167	167		
Age at surgery Mean (sd)	67.3 (12.0)	62.3 (10.9)	0.001	65.0 (12.8)	65.0 (10.9)	0.987	
Sex N Female (%)	25 (27%)	21 (26%)	0.900	46 (27%)	49 (29%)	0.808	
CCI N (%) 0 1 2 3 4 5 6 7 8 9	7(7.7%) 8(8.8%) 18(20%) 17(19%) 16(18%) 12(13%) 6(6.6%) 3(3.3%) 3(3.3%) 1(1.1%)	10(13%) 14(18%) 25(31%) 19(24%) 5(6.3%) 3(3.8%) 3(3.8%) 1(1.3%) - -	< 0.001	14(8%) 21(13%) 46(28%) 33(20%) 20(12%) 16(10%) 8(5%) 4(3%) 3(2%) 1(1%)	16(10%) 21 (12%) 47(28%) 35(21%) 17(10%) 18(11%) 5(3%) 8(5%) - -		0.966
ASA N (%) 0 1 2	6 (7%) 43 (47%) 42 (46%)	8 (10%) 45 (56%) 27 (34%)	0.200	17 (10%) 87(52%) 63(38%)	13(8%) 85(51%) 69(41%)	0.867	
BMI Mean (sd)	29.6 (6.07)	28.7 (5.58)	0.300	30.0 (6.32)	28.7 (5.20)	0.174	
eGFR Mean (sd)	70.9 (20.2)	78.4 (15.3)	0.023	73.8 (18.9)	73.1 (19.1)	0.870	
Tumor size (mm) Mean (sd)	31.2 (9.3)	37.3 (12.6)	< 0.001	33.1 (9.4)	34.6 (11.5)	0.394	
RENAL score N (%) 4 5 6 7 8 9 10 11	10(11%) 5(5.5%) 12(13%) 15(16%) 23(25%) 14(15%) 12(13%) –	8(10%) 6(7.5%) 13(16%) 21(26%) 11(14%) 15(19%) 5(6.3%) 1(1.3%)	0.300	16(10%) 9(5%) 24(14%) 27(16%) 39(23%) 28(17%) 25(15%) –	22(13%) 14(8%) 23(14%) 54(32%) 21(12%) 28(17%) 7(4%) 1(1%)	0.067	
ECOG Performance status N (%) 0 1 2 3	54(59%) 24(26%) 12(13%) 1(1.1%)	68(85%) 10(13%) 2(2.5%) –	< 0.001	118(71%) 34(21%) 14(8%) 1(1%)	123(73%) 31(18%) 15(9%) –	0.879	

To derive comparable outcomes data for the comparison, propensity score-weighted analyses (inverse probability of treatment weighting; IPTW) were carried out for two outcomes: time to local recurrence and time to metastasis. Sufficient data were available to carry out the propensity score matching for 80 patients undergoing RAPN and 91 patients undergoing PCA. There was only one death attributed to metastatic RCC and eight other deaths, which were insufficient to allow mortality analysis.

### Analytical Method

Potential outcome confounders (age, gender, tumor size, RENAL score, ECOG-PS, and age-adjusted Charlson comorbidity index) were included in the propensity score models. Scores were estimated by fitting a logistic regression model with stabilized IPTW, which was then used to derive pseudo-populations for analysis.

Survival analyses were based on time to local recurrence or metastasis. Patients who did not experience the event of interest or were lost to follow-up were right-censored at the last observed date. A Cox proportional regression model generated estimates of the hazard ratio.

**Fig. 3:**
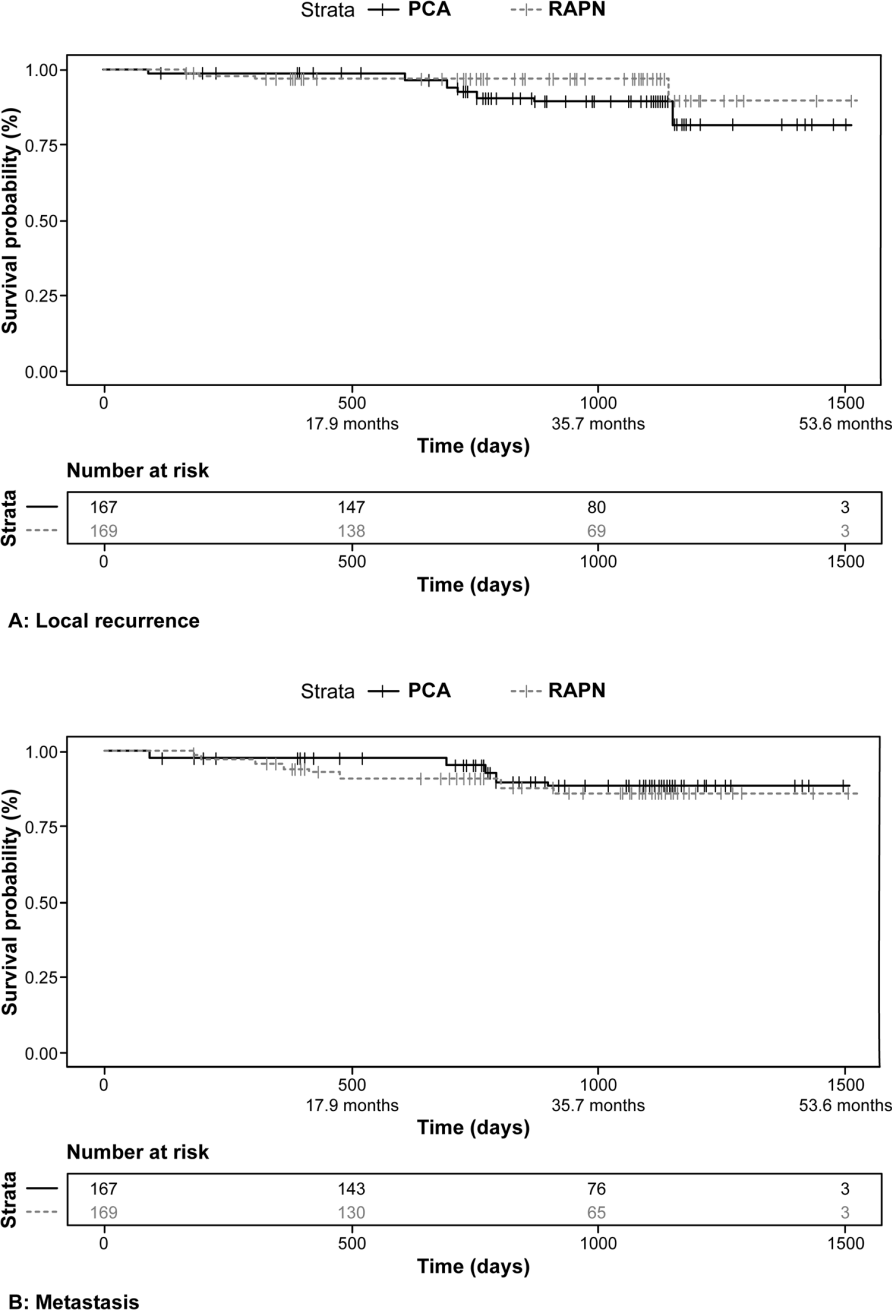
Time to event curves. Results of propensity score-matched analysis of patient databases. A = Local recurrence; B = Metastasis

The two pseudo-populations were well-matched (Table 2). Analysis demonstrated no significant differences between PCA and RAPN for either time to local recurrence (HR = 0.80; 95% CI = 0.34–1.85; *p* = 0.72) or time to metastasis (HR = 2.09; 95% CI = 0.69–6.26; *p* = 0.19) (Fig. 3). All analyses were conducted in R (R Foundation for Statistical Computing, Vienna, Austria).

Although the PCA group showed a trend toward earlier recurrence and later metastases, no statistically significant difference was seen for either of these two parameters. Thus, each group had identical transition probabilities for all oncological outcomes based on the 5-year event rates in the pseudo-populations. Mortality estimates for patients with metastatic disease were based on published 5-year survival data [19].

The 90-day postoperative complication rates for each treatment group were taken directly from the dataset: 2.5% in the RAPN group and 5.5% in the PCA group. Only complications with a Clavien-Dindo score of III or above were included, as lesser degrees would have been expected to impact costs and QALYs only modestly [20, 21].

The mean length of stay in the RAPN group was 1.90 nights. For PCA, 85/91 patients were treated as day cases, while the mean length of stay in the six remaining patients was 3.0 nights.

### Utilities

The utilities for stable and metastatic diseases were based on published results from the Netherlands [22]. The utility for recurrent disease was assumed to fall midway between stable and metastatic disease, in line with the approach used in a recent economic analysis [13]. The utility for postoperative complications was taken from Casciano et al. [23].

### Costs

All costs were expressed as Euros (€), using an exchange rate of DKK 1 = €0.134.

Procedural costs for RAPN were based on the 2024 tariff for Diagnosis-Related Group (DRG) 11MP07 (*operations on kidney, renal pelvis, and ureter, malignant disease, patient aged* > *18, with robot*): €15,293. No specific DRG for PCA is currently approved in Denmark. The estimated per-procedure cost of €7,427 was consequently based on local resource use assessments of staff costs, length of stay, and costs of major single-use equipment.

## Results

Results (Table 3) demonstrate a near equality of efficacy outcome: both interventions yield a similar discounted 5-year QALY gain (3.323 vs 3.315). Over 5 years, total costs associated with PCA were 32% lower than those incurred in the RAPN group (€18,795 vs €27,520). This suggests that PCA marginally dominates RAPN, although the minimal incremental QALY makes this an uncertain conclusion. More informative in this situation is the net monetary benefit (NMB), which places the assumed value of the QALY gain alongside the difference in resource use in a single metric. A positive value for the NMB implies a cost-effective treatment at the stated willingness-to-pay threshold. The base case demonstrates an NMB of +€9,045 for this model, at a willingness-to-pay threshold of €40,000/QALY, confirming that PCA is a cost-effective interventio**n.**

**Table 3:** Results of base case analysis—5-year time horizon; costs and benefits discounted at 3% per annum, with a willingness to pay = €40,000/QALY

Treatment group	Absolute		Incremental		ICER	NMB
	*Cost*	*QALYs*	*Cost*	*QALYs*		
RAPN	€27,520	3.315				
PCA	€18,795	3.323	€-8,725	0.008	Dominant	€ 9,045

ICER = Incremental cost-effectiveness ratio; NMB = Net monetary benefit

**Fig. 4:**
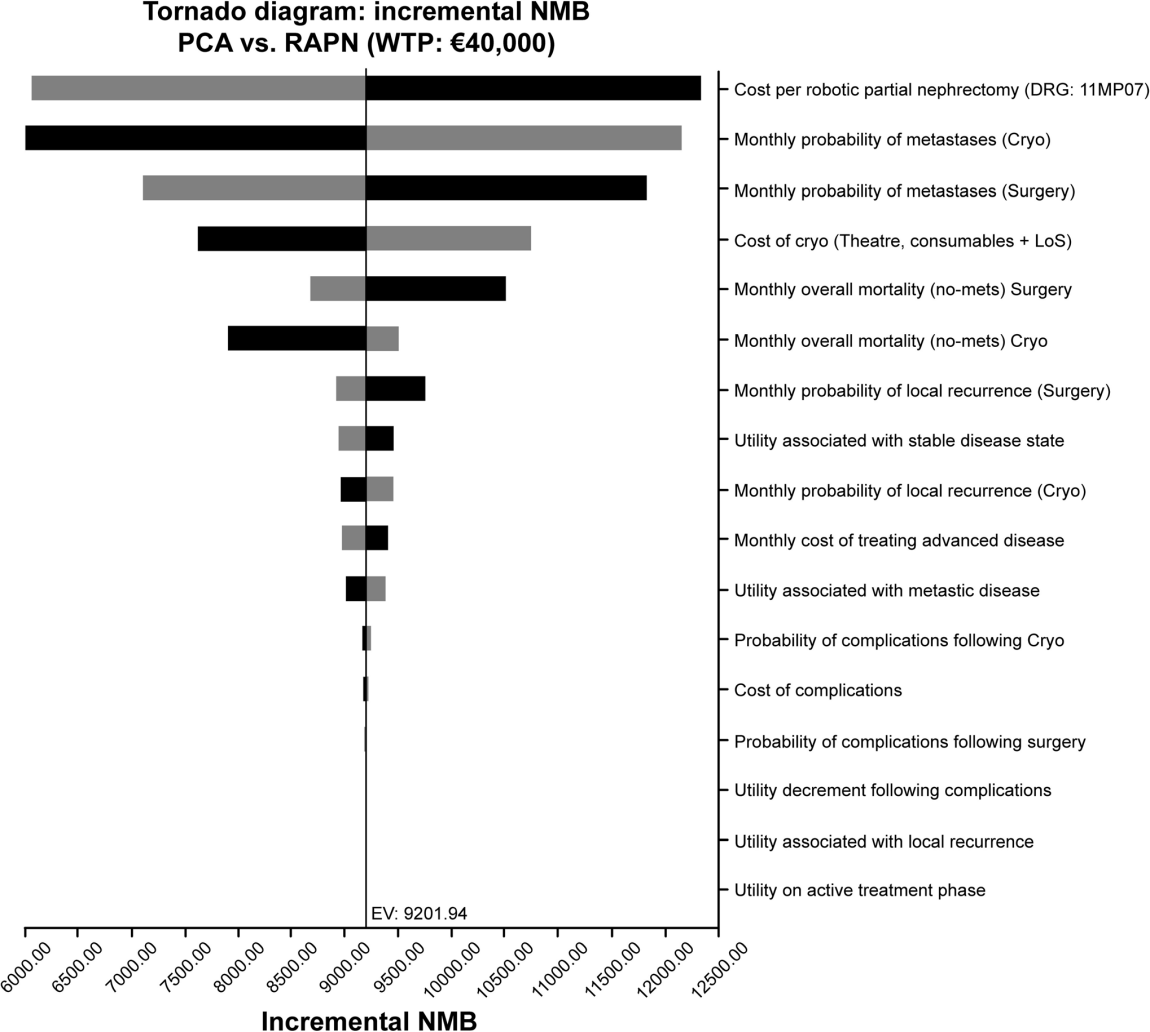
Tornado plot showing the impact of the deterministic sensitivity analysis on net monetary benefit. Top 17 parameters listed based on the magnitude of effect on NMB *Black bars* = *NMB based on high parameter estimate; Gray bars* = *NMB based on low parameter estimate*

Deterministic sensitivity analysis (Fig. 4) shows that the NMB is most sensitive to variations in the probability of metastases for the two treatment strategies, with procedural cost parameters also significantly affecting the outcome. Other factors, such as local recurrence risk and the cost of downstream treatments, are less essential drivers of NMB. All explored ranges showed positive NMB, indicating robust cost-effectiveness for PCA.

## Discussion

This study uses prospective real-world data from two Danish centers to demonstrate that PCA has comparable oncological outcomes to RAPN in patients with stage T1 RCC. Using these data in a cost-effectiveness model yields near-identical health benefits over five years. However, the overall cost of PCA treatment is lower, making PCA the economically dominant treatment over RAPN. The finding of a positive NMB indicates that PCA represents a more efficient use of resources and is consequently the preferred choice from a health economic perspective.

The lower costs for PCA are driven by the index procedure's costs—principally lower theater costs and shorter postoperative length of stay. The 90-day rate of postoperative complications at Clavien-Dindo grade III and above was non-significantly higher in the PCA group vs the RAPN group (5.5% vs 2.5%). Analysis of the complication rates in this dataset has been previously published [17], with the authors noting that the observed rates in the PCA group are higher than rates seen in an earlier retrospective case series that also used the Clavien-Dindo classification [24]. This appears to reflect significantly different durations of postoperative follow-up for complications in the two studies (90 days vs 30 days). In the current dataset, the principal driver of the excess severe complication rate for PCA was six cases of delayed abscess formation diagnosed after 30 days. In the Schmit et al. case series, only one abscess occurred out of 398 cases analyzed [24]. When a 30-day limit was applied to the current dataset, the observed complication rates were similar between the two studies. Additionally, sensitivity analysis shows these costs are relatively unimportant determinants of overall health economic outcomes [Fig. 4]. Consequently, this is unlikely to have influenced the result of the current analysis.

Previously published health economic analyses exist comparing nephron-sparing surgery with percutaneous localized treatments [13, 14, 25–29], although in the absence of randomized controlled trials, these are typically derived from retrospective case series. The conclusions of a recent literature-based cost-utility analysis carried out by Wu et al. were very similar to those of our analysis, with 5-year efficacy outcomes being nearly identical between the two groups (3.68 vs 3.67 QALYs). Costs in the PCA group were 23% lower ($20,491 vs $26,478 ~ €18,278 vs €23,618) [13]. It should be noted that the primary data source in Wu et al.’s model came from patients treated before 2011, and the PN group pertains to open surgery instead of laparoscopic or robot-assisted procedures. Additionally, although propensity score matching was carried out, limited information was available to characterize the tumor complexity, so it is uncertain how well-matched the two treatment groups were [13].

In a cost-consequences analysis, Garcia et al. demonstrated a 36% cost difference favoring PCA vs RAPN in a single-center setting in Brazil ($12,435 vs. $19,399 ~ €10,545 vs €16,450) [14]. This analysis was based on a relatively recent dataset, but the data were not corrected for baseline differences in the treated populations. As our study showed, patients treated with PCA tend to be older, with more comorbidities and higher RENAL scores than those treated with RAPN. The absence of matching in the study by Garcia et al. introduces uncertainty to the quantitative results [14].

Although other, older economic analyses exist, they generally reflect technologies and outcomes that are not representative of current practice and which, consequently, are of limited relevance to the current research question and clinical practice [25–29].

### Limitations

Patient data for this analysis were drawn from two University Hospitals in Denmark. All patients undergoing PCA were treated at a single hospital with the appropriate experience and expertise, while both centers undertook RAPN. This imbalance may yield relevant organizational issues that we have been unable to consider in our analysis.

As discussed, baseline differences in the two patient populations also present a source of potential bias. Patients treated with PCA tended to be older and had a higher RENAL score, reflecting more advanced tumors with higher case complexity and, consequently, surgical and oncological risk. Furthermore, patients in the PCA group had a greater prevalence of life-limiting non-renal comorbidities, as evidenced by a higher Charlson comorbidity index. We undertook a propensity score weighting approach to generate matched pseudo-populations to minimize the impact of these differences on the clinical outcome results. However, even though we achieved a high level of matching for the parameters under consideration, it is possible that there remained unmatched confounders.

Additionally, the analysis included both T1a and T1b tumors. However, subgroup analysis by stage was not performed due to limited statistical power, especially within the cryoablation subgroup. This should be considered when comparing our findings to studies that included only T1a tumors, as differences in tumor stage may influence outcomes and generalizability.

The approach for assigning costs to the treatment options is a limiting factor to the analysis. For RAPN, a specific DRG is identified within the Danish tariff (11MP07) with a value of €15,293. There is no specified DRG for PCA, so a bottom-up estimate was used based on known materials costs and clinician-estimated healthcare resource use. In the absence of contemporaneously collected resource use data, it is possible that either estimate could be an inaccurate representation of the actual costs. Infrastructure costs were not captured, nor were costs for potential additional procedures, such as inserting a JJ stent before PCA of a complex tumor, included. However, the relative costs of RAPN and PCA are consistent with the values used in other published comparisons [14, 28, 29].

Structural issues within the model may have impacted the results. A 5-year time horizon was used to maintain the modeled outcomes within the evidence’s timeframe, avoiding the need for extrapolation, which could have introduced bias. Given that most of the between-group differences observed in the model accrue in the perioperative period, with minimal differences in long-term oncological outcomes expected, extrapolation would have been unlikely to produce a more informative result.

## Conclusion

The present study reported comparable oncological outcomes following PCA and RAPN, utilizing prospectively collected real-world data from patients with RCC T1, leading to near-identical QALYs for the two treatment options. Additionally, a 32% cost reduction was found for PCA compared to RAPN. This study suggests that PCA may offer cost savings for RCC T1 patients without compromising oncological outcomes, making it a cost-effective treatment option over a 5-year time horizon.
